# Nanostructural Differentiation and Toxicity of Amyloid-β25-35 Aggregates Ensue from Distinct Secondary Conformation

**DOI:** 10.1038/s41598-017-19106-y

**Published:** 2018-01-15

**Authors:** Yongxiu Song, Ping Li, Lei Liu, Christian Bortolini, Mingdong Dong

**Affiliations:** 1 Institute for Advanced Materials, Jiangsu University, Zhenjiang, China; 2 National Center for Nanoscience and Technology (NCNST), No. 11, BeiyitiaoZhongguancun, Beijing, China; 3 Interdisciplinary Nanoscience Center (iNANO), Aarhus University, Gustav Wieds Vej 14, Building 1590, Aarhus C, Denmark

## Abstract

Amyloid nanostructures are originated from protein misfolding and aberrant aggregation, which is associated with the pathogenesis of many types of degenerative diseases, such as Alzheimer’s disease (AD), Parkinson’s disease (PD) and Huntington’s disease. The secondary conformation of peptides is of a fundamental importance for aggregation and toxicity of amyloid peptides. In this work, Aβ25-35, a fragment of amyloid β(1-42) (Aβ42), was selected to investigate the correlation between secondary structures and toxicity of amyloid fibrils. Furthermore, each aggregation assemblies show different cell membrane disruption and cytotoxicity. The structural analysis of amyloid aggregates originated from different secondary structure motifs is helpful to understand the mechanism of peptides/cell interactions in the pathogenesis of amyloid diseases.

## Introduction

The aggregation of amyloid peptides is closely related to the pathogenesis of many kinds of degenerative diseases^1–3^. A viable route to study the pathogenesis of these amyloid diseases is to investigate cell impairment originated from amyloid peptide aggregates^4–8^. The secondary structure of peptides determines the morphology of amyloid aggregates, which implies that amyloid aggregates play an important role on cell interruption^9^. In the abnormal assemblies of peptides, the change of physicochemical properties of aggregations is strongly related to secondary structure of misfolded proteins or even the conformation rearrangement, resulting in amyloid-related diseases^9,10^. For instance, the cross β-structure of prion proteins in infectious aggregates induces the conformation conversion from α-helical to cross β-structure, which does facilitate the amyloid-like fibril formation resulting cytotoxic^9^. The secondary structures of the amyloid aggregates have been extensively explored by a diversity of diffraction techniques such as X-ray scattering and electron diffraction^11^ and solid-state NMR spectroscopy^12,13^. However, these methods can only supply average structural information. Recently, scanning tunneling microscopy (STM) has been utilized to explore the molecular assembled structure of amyloid peptides, which can reveal the mechanistic insight of peptide assembly at submolecular level^14,15^. Although STM is a powerful tool, amyloid aggregates at nanoscale are still needed to reveal cellular level membrane interruption. On the other hand, Atomic force microscopy (AFM) is feasible methods to reveal the self-assembly process, and provide the dynamics information of amyloid assembly^16–21^, which can reveal the mechanism of aggregation. In this work, we focus on determination and analysis of peptide secondary structural effect on morphology and cytotoxicity of amyloid aggregates by high resolution AFM combined with circular dichroism (CD) spectra and cell assay. Aβ25-35 is chosen as the model of Aβ42 since it is generally considered as the biologically active region of Aβ, it also represents the shortest fragment able to exhibit large β-sheet aggregated structures and is considered as the most toxic peptide fragment derived from APP^22,23^. We found that the aggregation of Aβ25-35 is strongly affected by ions in solution. The kinetics of amyloid fibrillization and the structural transition were examined by thioflavin T (ThT) fluorescence and CD, respectively. In water solution, the secondary structure different from β-sheet secondary structure leads to flat fibrils. Amyloid peptides convert into twisted β-sheet secondary structure when employing a phosphate buffer solution instead of Milli-Q water. These two amyloid aggregates present different adhesion force map based on AFM measurement, which could be ascribed to distinct packing of secondary structures. Furthermore, based on our cytotoxicity study of Aβ25-35, twisted β-sheet fibril structure was observed to be more toxic compared with the flat fibrils. The cell membrane interruption and the cell affinity of twisted β-sheet fibrils are much stronger in comparison with the flat fibrils. Two kinds of amyloid aggregates originated from different secondary structures are linked to cytotoxicity during the interaction with the cell membrane, which is in general related to the pathogenesis of amyloid disease.

## Results and Discussion

### The structure and the secondary structure detection of Aβ25-35

**Figure 1:**
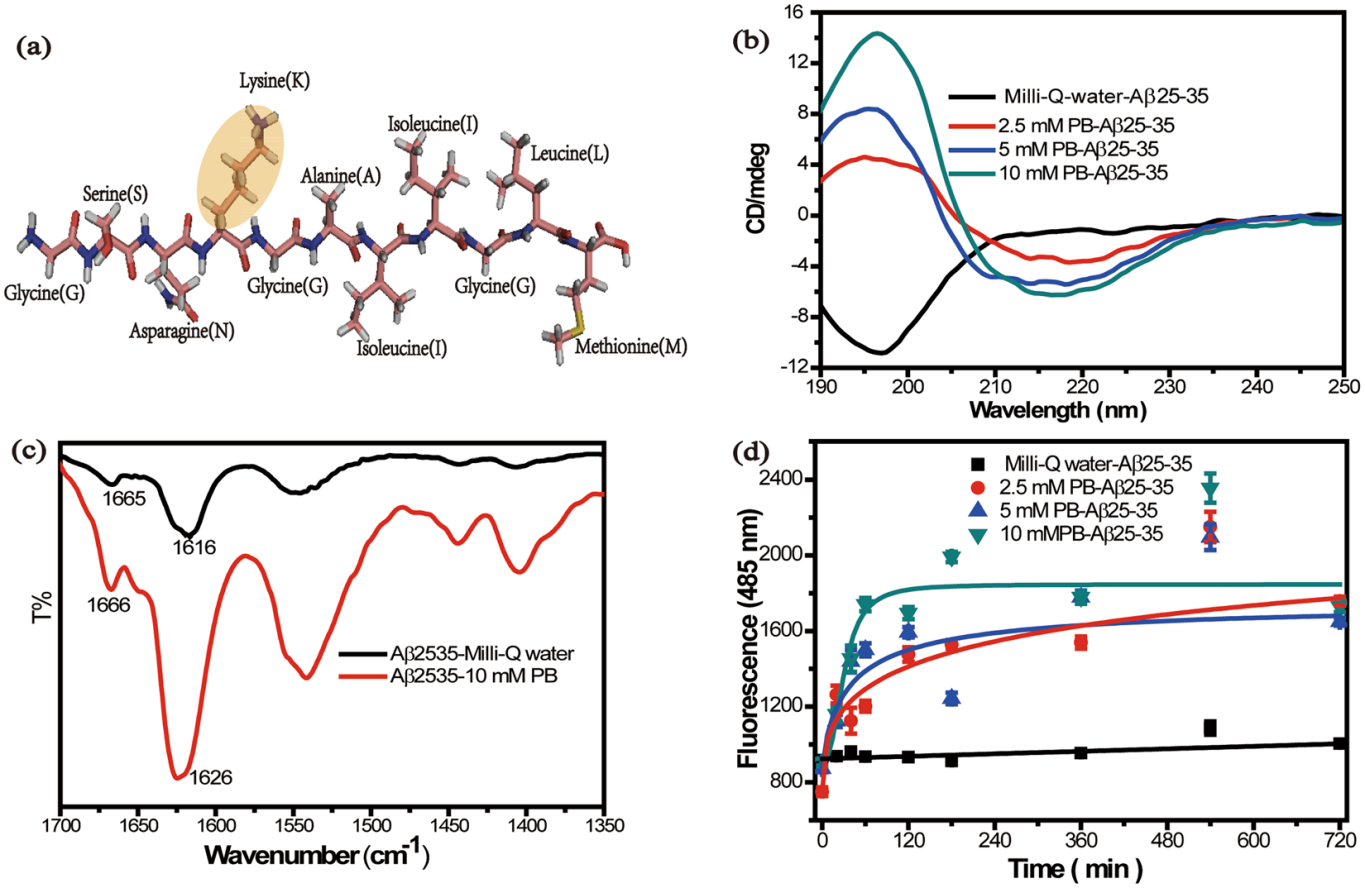
(**a**) Molecular model of Aβ25-35 (the light yellow oval indicates Lysine). (**b**) Circular dichroism spectra of Aβ25-35 (100 μM) after 12 h incubation at 37 °C at 0, 2.5, 5 and 10 mM of PB. (**c**) ATR-FTIR spectrum of Aβ25–35 (100 μM) in Milli-Q water and PB solution. (**d**) Aggregation of Aβ25-35 as followed by ThT assay.

Aβ25-35 originates from residue 1–42 of amyloid β peptide^24,25^. This segment consists of 11 amino acids (Fig. 1(a)). Lysine (K) has a positive charge^26^, as shown in the light yellow oval of Fig. 1(a). The secondary structure of the peptide was determined by circular dichroism (CD)(Fig. 1(b)). CD spectra of Aβ25-35 in Milli-Q water displays a negative minimum at 197 nm and a smaller positive maximum at 218 nm (seeing Fig S1). Interestingly, the CD spectra of Aβ25-35 in PB solution (2.5 μM, 5 μM, 10 μM) was different compared to the one in water, which exhibited a negative peak at 218 nm and a positive peak at 197 nm suggesting the presence of β-sheet structures^27^. In addition, the intensity at 218 nm (or 197 nm) was found to be directly proportional to the concentration of PB, which suggests that the phosphate ion can affect the conformation of β-sheet structure. In order to thoroughly identify the secondary structure of Aβ25-35 in diverse solutions, ATR-FTIR experiments were performed, as shown in Fig. 1(c). Aβ25-35 in PB solution exhibited two main absorbance peaks at 1626 cm^−1^ and 1666 cm^−1^, indicating the typical secondary conformation of β-sheet structure^28^. However, Aβ25-35 in Milli-Q water presented two absorbance peaks at 1616 cm^−1^ and 1666 cm^−1^ based on ATR-FTIR, which present the regions of amide I band^29^. Besides, Dichroweb CD analysis^30–32^(Table 1) showed that the amount of unordered structure decreased by adding the phosphate ions into Milli-Q water gradually. To further detect amyloid peptide secondary structure, ThioflavinT(ThT) fluorescence has been widely used to monitor amyloid peptide fibrillation kinetic profiles by displaying fluorescence as an effect of the binding to β-sheet domains on amyloid aggregates^33^. As shown in Fig. 1(d), the curves represent the peptide fibrillation process. We can observe that Aβ25-35 in PB solution has a very strong fluorescence in this assay and the aggregation of Aβ25-35 reached the plateau phase of fibrillation fast when the concentration of PB solution was 10 mM, indicating that the phosphate ion promoted the formation of β-sheet structure of Aβ25-35 and altered the aggregation of Aβ25-35. Additionally, the aggregation of Aβ25-35 in Milli-Q water showed little fluorescence signal further indicating that the secondary structure of Aβ25-35 in Milli-Q water was not a β-sheet structure^33^. By combining our findings with CD and ATR-FTIR measurements. We can conclude that the phosphate ions strongly influence the secondary structure of Aβ25-35.

**Table 1:** Secondary structural components of Aβ25-35 fibrils, obtained visa CD data.

	MQ-water-Aβ25-35	2.5 mM PB-Aβ25-35	5 mM PB-Aβ25-35	10 mM PB-Aβ25-35
alpha-helices	9	15	27	29
beta-sheets	32	34	29	36
unordered	36	30	27	21
turns	23	21	17	14

### Fibrillation of Aβ25-35

**Figure 2:**
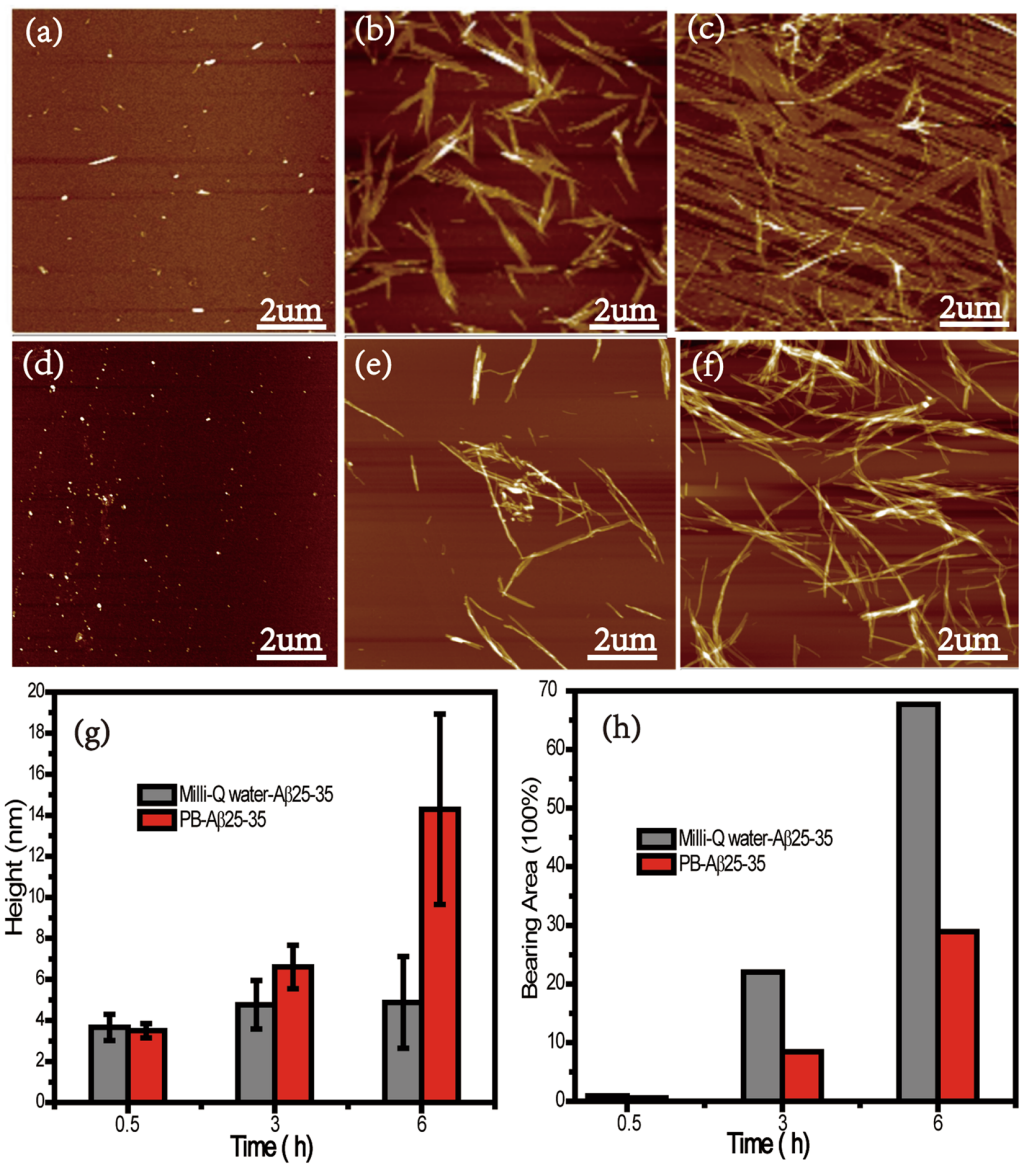
AFM images of Aβ25-35 in water at (**a**) 0.5 h (**b**) 3 h (**c**) 6 h; AFM images of Aβ25-35 in PB at (**d**) 0.5 h (**e**) 3 h (**f)** 6 h. (**g**) The histogram with Gaussian fitting of the height of Aβ25-35 aggregates. (**h**) The histogram of the height of the bearing area of Aβ25-35.

**Figure 3:**
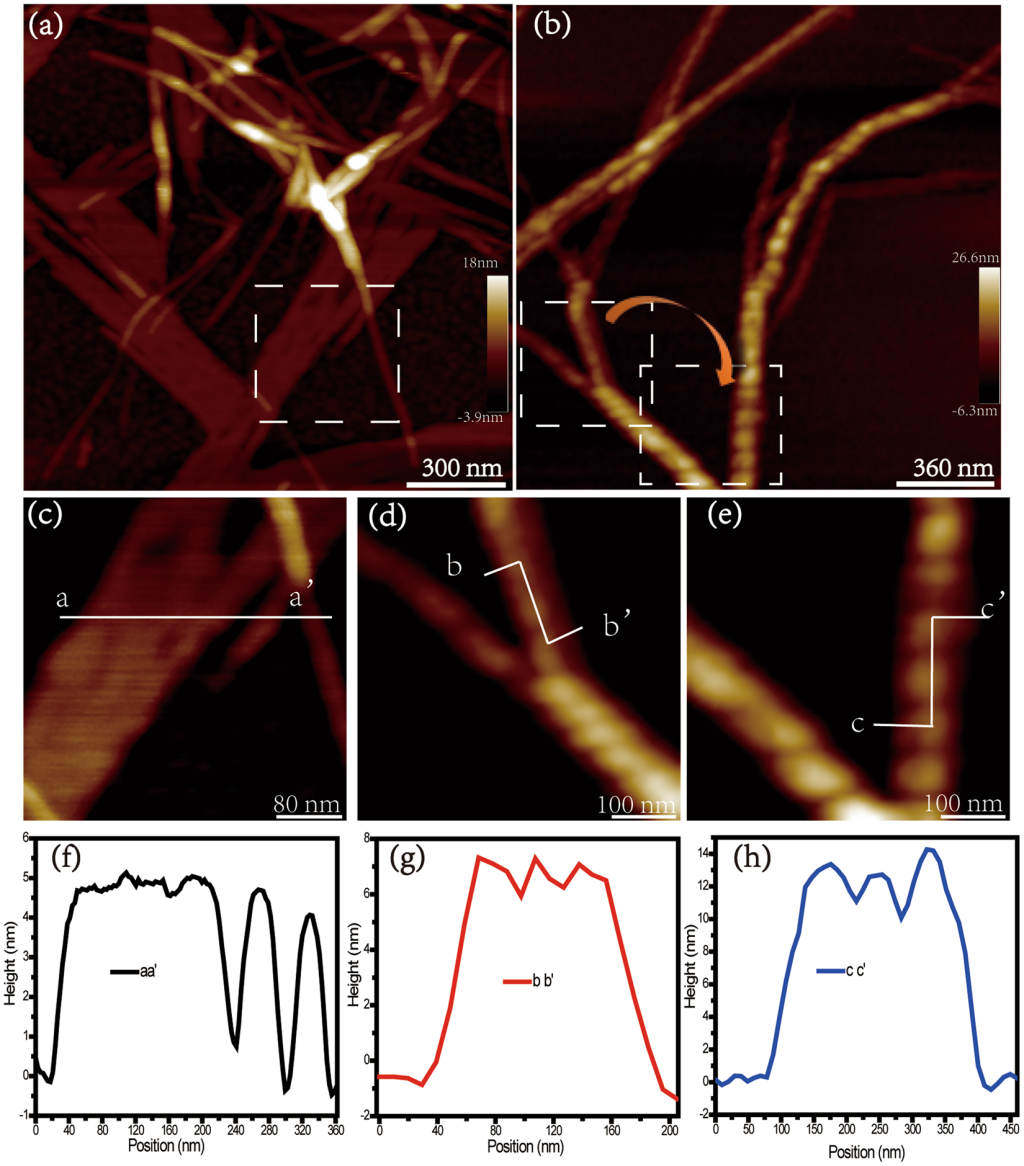
AFM morphology images of Aβ25-35 in Milli-Q water (**a**) or PB (**b**) at 12 h; AFM morphology images of Aβ25-35 nanostructure in Milli-Q water (**c**) or PB (**d**,**e**). Height of line profiles of Aβ25-35 fibrils are indicated by aa’ in (**f**), bb’ and cc’ in (**g**) and (**h**), respectively.

The formation of β-sheet structures promoted by phosphate ions probably lead to different aggregations of Aβ25-35 from that in Milli-Q water. The morphology of the nanostructures can be assessed by using Atomic Force Microscopy (AFM). AFM images taken during aggregations of Aβ25-35 in PB solution or Milli-Q water are shown in Fig. 2(a–f). At 0.5 h, only some oligomeric species were found with the height of 3.6 ± 0.6 nm (Fig. 2(a)) and 3.5 ± 0.4 nm (Fig. 2(d)), respectively. Morphological changes were observed after 3 h incubation and proto-fibers were obtained 4.8 ± 1.2 nm and 6.6 ± 1.1 nm tall (Fig. 2(g), histogram) in Milli-Q water and PB solution, respectively. The proto-fibers in Milli-Q water are shaped like bamboo leaves while the short linear fibrillar structures were present in PB solution. These peculiar morphological changes became more evident after incubation for 6 h, thin flat film with the diameters of 4.9 ± 2.2 nm were observed in Milli-Q water. At the same time, a high density of long mature fibrils with diameters of 14.3 ± 5.8 nm appeared in PB solution. Finally, mature fibers were formed after incubation for 12 h (Fig. 3(a) and (b)). Furthermore, AFM images of Aβ25-35 on SiO_2_ and glass substrates, the size distribution and turbidity of Aβ25-35 confirmed the fiber formed in bulk Milli-Q water (shown in Fig S2). The bearing area of the height of Aβ25-35 aggregates at different time points was shown in Fig. 2(h). Together with the height distribution at different time points and the corresponding morphology, a coherent picture of the self-assembly process emerges.

**Figure 4:**
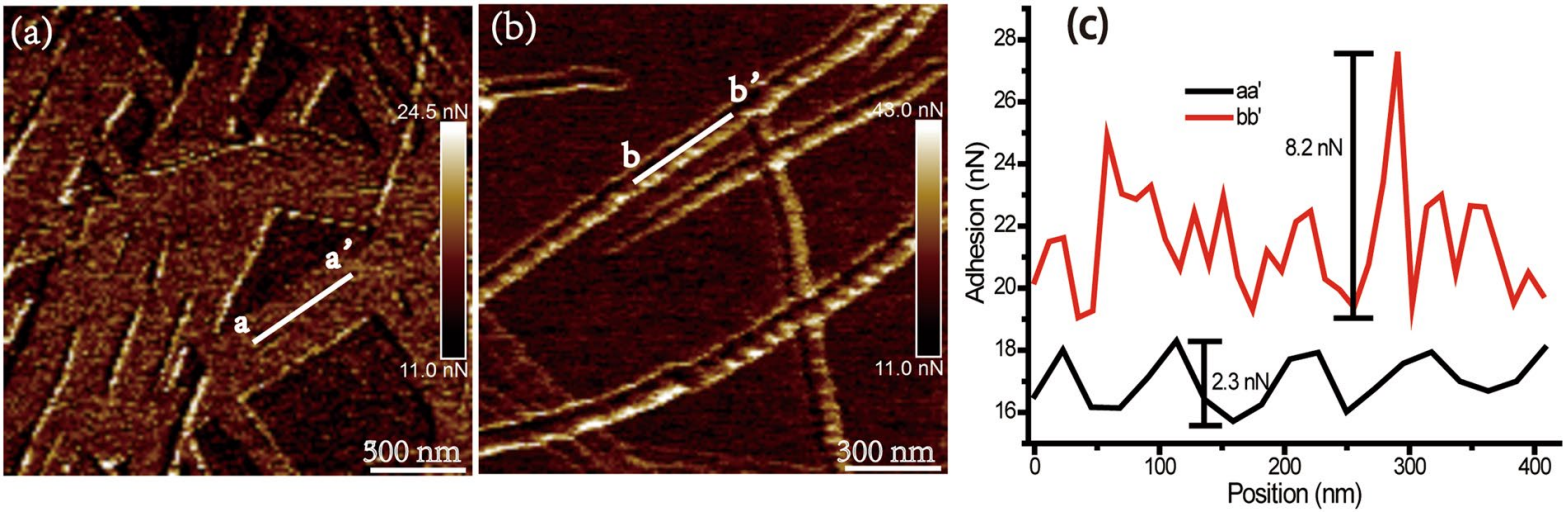
Adhesion map of Aβ25-35 amyloid aggregates. (**a**) Adhesion map of flat assembled nanostructure of Aβ25-35 in Milli-Q water. (**b**) Adhesion map of twisted fibril of Aβ25-35 in PB solution. (**c**) Adhesion force line profiles of Aβ25-35 assembled nanostructure are indicated by aa’ in (**a**) and bb’ in (**b**), respectively.

From the fibrillation process of Aβ25-35 amyloid peptide in Milli-Q water and PB buffer solution, the height of mature fibrils increased after the end of plateau phase of fibrillation (ThT assay in Fig. 1(d)), which were identified in Fig. 2(b,c,e,f). Then, we systematically analyzed the detailed structure information of mature fibrils in Milli-Q water and in PB buffer solution by using high-resolution AFM. The two different types of nanostructures assembled from Aβ25-35 were observed, namely the flat film and the twisted fibrils (Fig. 3(a) and (b)). The height value of flat film and fibril in Milli-Q water was similar and approximately 5.0 ± 1.0 nm (line profile of aa’ in Fig. 3(c)), indicating that the film is composed of fibrils. Combining the morphology analysis of Aβ25-35 aggregates with the incubation of 6 h and 12 h in Milli-Q water (Fig. 2(c) and Fig. 3(a)), we proposed that the fibrils continue to grow on the surface of the film. In case of twisted fibrils formed in PB buffer solution, the height of a single fibril was approximately 11.0 ± 1.1 nm. There are two twisted structures in PB buffer solution (Fig. 3(b)). The heights of fibrils were about 7 nm (Fig. 3(d)) and 13.5 nm (Fig. 3(e)). The periodicity of these two twisted structures was estimated to be about 30.5 nm (Fig. 3(d)), and 78.1 nm in Fig. 3(e). Our hypothesis is that the twisted nanostructure in Fig. 3(e) was formed by fibers in Fig. 3(d). The height distribution of the twisted structure may be formed by two fibers (line profile of cc’ in Fig. 3(e)) twisted. Furthermore, the adhesion force maps of amyloid aggregates were obtained (Fig. 4). The flat fibril film assembled from amyloid peptide displayed the relative “homogeneous” adhesion force on the surface, compared to the twisted fibril structure of amyloid peptide in PB solution. The fluctuation of adhesion force is approximately 2 nN in the case of the flat film (Fig. 4(a)), while it reached to 8 nN in the twisted fibril structure (Fig. 4(b)), which can be attributed to the different molecular packing in the assembled structure. The distinct secondary conformations result in various assembled structures in which the residues of peptides may expose on the fibers’ surface different functional groups of their side-chains. Charged residue such as Lysine might be more exposed in the case of twisted fibril than it does in the flat film. The surface adhesion properties can be identified by adhesion force map, which is also an important factor for the affinity with the cell membrane.

### Detection of the cytotoxicity and cell membrane disruption of amyloid aggregates

**Figure 5:**
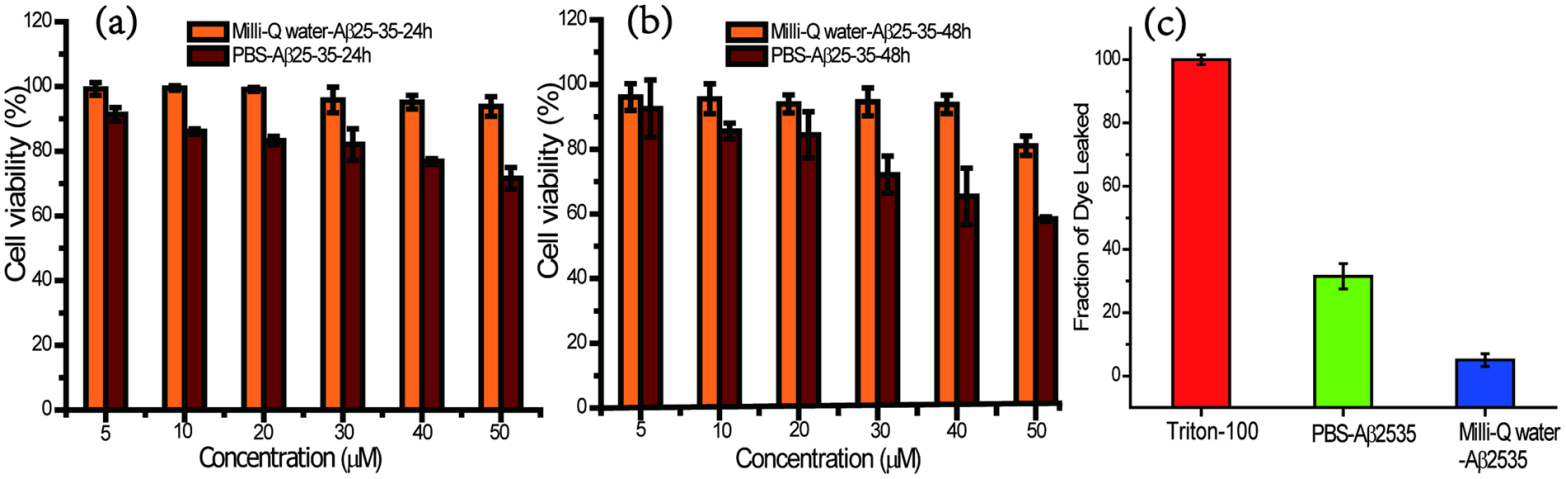
Cytotoxic effects of Aβ25-35 in Milli-Q water or PBS buffer. *In vitro* viability of SH-SY5Y treated with Aβ25-35 at 5, 10, 20, 30, 40 or 50 μM for 24 h (**a**) and 48 h (**b**) incubation, respectively (n = 6). Cell viability was determined using the CCK-8 Kits and the absorbance was detected at 450 nm. (**c**) Dye leakage from DOPC vesicles induced by Aβ25-35 aggregates in different condition.

**Figure 6:**
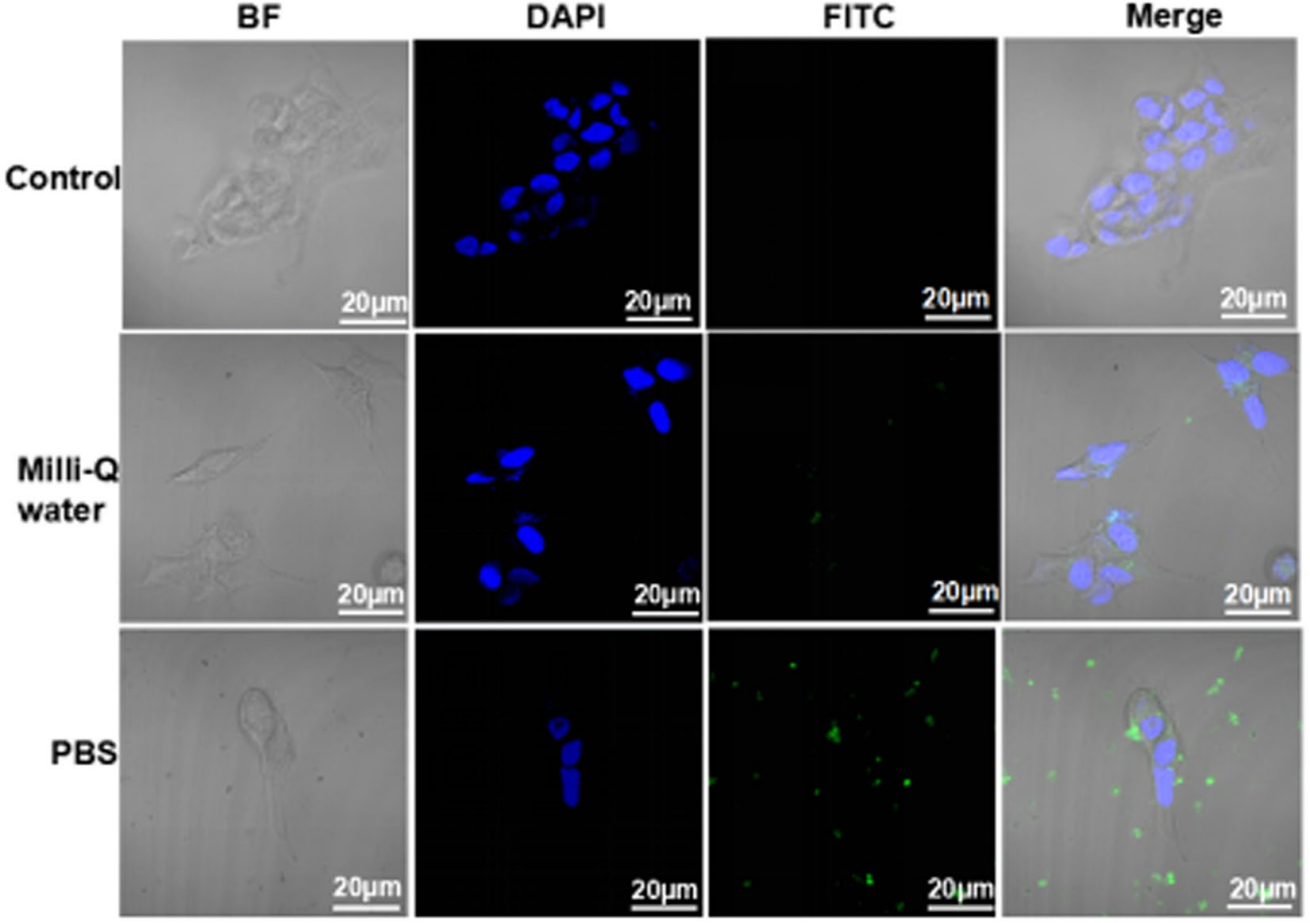
Interaction between SH-SY5Y cells and amyloid peptide labeled with FITC in PBS buffer and Milli-Q water. Cells were incubated with fluorescent dyeAβ25-35 at the same concentration of 40 μM for 24 h at 37 °C and then observed by confocal microscopy. The excitation/emission wavelengths were 488/530 nm for fluorescent Aβ25-35 amyloid peptide and 358/461 nm for DAPI.

To explore the cytotoxicity of Aβ25-35 with different secondary structure motifs in different solutions, SH-SY5Y cells were used to assess the cell viability. Figure 5(a) and (b) show the *in vitro* viability of cell after 24 h and 48 h incubation with Aβ25-35 amyloid peptide at a concentration ranging from 0 to 50 μM. Cytotoxicity of Aβ25-35 in PBS solution increased with increasing concentration of amyloid peptide and the incubation time. When the cells were incubated with 50 μM Aβ25-35 in PBS buffer for 24 h and 48 h, there were only 72.8 ± 3.7% and 59.4 ± 1.0% cells alive, respectively. As opposed to the case of PBS incubation, there were little cells apoptosis caused by Aβ25-35 amyloid peptide in Milli-Q water with increasing exhibited cracking features, and cells were covered by Aβ25-35 amyloid peptide with micrometer size (shown in Fig S5). These results are clear indication of a higher cytotoxic effect of Aβ25-35 in PBS solution with β-sheet conformation rather than Aβ25-35 in Milli-Q water with distinct secondary structure conformation for SH-SY5Y cells after treatment. To further investigate the cytotoxicity of Aβ25-35 related to its the secondary structure, we proposed the membrane disruption possibly induce to impairing the cell. The hemolysis experiment is aiming to explore the membrane disruption of Aβ25-35 aggregates in the cell-like system (shown in Fig S6), and the dye-release assay in liposome which is a typical approach to investigating the ability of membrane disruption of nanomaterials was also performed (shown in Fig. 5(c)). Aβ25-35 aggregates in PBS solution induced more membrane disruption than the one in water. We could verify the membrane disruption of Aβ25-35 peptide aggregates, which is consistent with the results we obtained by hemolysis experiment. Consistent with the cell viability assay, Aβ25-35 in PBS buffer was found to be more toxic than that in Milli-Q water. We then assessed the interaction between SH-SY5Y cells and Aβ25-35 amyloid peptide aggregates by fluorescence microscopy. Figure 6 displays the fluorescent images of SH-SY5Y cells with Aβ25-35 amyloid peptide in PBS buffer and Milli-Q water. Cell structure and cell core can be identified by bright field (BF) mode and fluorescence mode with 4′, 6′-diamidino-2-phenylindole (DAPI). The interaction between the cell and amyloid aggregates was observed by Merge mode. We observed that amyloid aggregates in Milli-Q water rarely interact with the cell membrane, while aggregates in PBS buffer solution can attach on the cell membrane. Fluorescent peptides (FITC labeling of Aβ25-35 do not change the secondary structure of the peptide, shown in Fig S6) can assemble into micro size aggregate and bind to the surface of cell membrane, but not enter into the cells, which gives the mechanistic insight of amyloid aggregates interrupting the neuron cell and also provide us with further insights on the peptide secondary structural effect of amyloid aggregates on the cytotoxicity.

In this work, we investigated the peptide secondary structural effect of amyloid aggregates on assembled nanostructure and cytotoxicity systematically. This study demonstrated that amyloid aggregates based on β-sheet secondary conformations prefer to bind to SH-SY5Y cell membrane in case of Aβ25-35 amyloid peptide. In the adhesion map of amyloid twisted structure, a big fluctuation of adhesion force on the surface of nanostructure was observed. The different interaction between amyloid aggregates and cell membrane is closely linked to the cytotoxicity.

## Conclusions

In summary, the self-assembly of amyloid peptide Aβ25-35 in different conditions were characterized by high resolution AFM, adhesion force mapping, CD spectra and ATR-FTIR. The cell assay was used to analyze the secondary structural effect of amyloid aggregates on assembled nanostructural cytotoxicity. The results showed that β-sheet secondary structure of Aβ25-35 peptide resulted in the assembled twisted fibril, while the one with different secondary structure assembled as a flat film. Distinct secondary conformation induced a big fluctuation of adhesion force on the surface of nanostructure and different cytotoxicity to SH-SY5Y cells. We assume this distinct secondary conformation may be attributed to the positive charge (Lysine) of peptides exposed on aggregates’ surface, which facilitates the interaction between the peptide aggregates and the cell membrane with negative charge^34,35^. Our work can open an approach towards analyzing and exploring the mechanistic insight of amyloid peptide assembled structure and finding a way to link to the functionality. Our study provided insights into the secondary structural effect of amyloid aggregates on morphology and cytotoxicity, which could be beneficial for understanding the pathogenesis of amyloid diseases.

## Methods

### Preparation of Amyloid Fibrils

1 mg Aβ25-35 (amino acid sequence: NH_2_-GSALGAIIGLM-COOH; American Peptide Company, USA) was dissolved in 1 mL 1,1,1,3,3,3-hexafluoro-2-propanol (HFIP; Tokyo Chemical Industry, Japan), and subsequently sonicated for 5 seconds and vortexed 3 times for 5 seconds. Then the solution was kept in a thermo-shaker (PHMT, Grant Instruments, England) for 24 h at 350 rpm min^−1^ at 25 °C. After that, the solution was stored in the freezer at −20 °C before use. 35 μL of Aβ25-35 HFIP solution were transferred into a 1.5 mL centrifuge tube, and the solvent was removed by storing it in a vacuum drying oven (Jinghong Co., Ltd., China) for 1 hour at 25 °C. Afterwards, the peptide film in the tube was dissolved in Milli-Q water or phosphate buffer solution to the final concentration of 100 μM. Finally, the peptide solution was incubated on the thermo-shaker for 12 hours at 350 rpm min^−1^ and 37 °C.

### Atomic Force Microscopy

20 μL of incubated peptide solution were deposited onto the freshly cleaved mica surface and air-dried for 10 min, then the residues was removed. The sample was rinsed once with Milli-Q water and dried in ambient condition before measurement. All AFM height images were recorded under ambient condition with a commercial AFM MultiMode VIII (Bruker, Santa Barbara, USA) in a PeakForce-tapping mode with an ultrasharp silicon probe with the spring constant of 26 Nm^−1^. Adhesion map images were recorded in a PeakForce QNM mode with an ultrasharp probe with the spring constant of 200 Nm^−1^. The AFM images were taken on 10 different places.

### Circular Dichroism (CD) spectroscopy

CD spectra measurements were performed on a spectropolarimeter (JASCO, Hachioji City, Japan) with a model No. PTC-348W1 (JASCO) with Aβ25-35 peptide solution both in Milli-Q water and phosphate buffer (pH = 7.15) solution at the concentration of 100 μM. All experiments were performed at 25 °C and a 0.1 cm quartz cuvette was used with a spectral region of 190–250 nm and a scan speed of 50 nm min^−1^. Besides, the slit-width was set at 1 nm. For all samples, the signal of Milli-Q water and phosphate buffer solution (pH = 7.15) were subtracted as the baseline. The sample volume for each CD measurement was 300 μL. The HT voltage was no more than 400 V for all samples test. Each experiment was performed in triplicate.

### Fourier Transform Infrared Spectra (ATR-FTIR)

The secondary structure of Aβ25-35 was analyzed on a Nicolet iS5 FT-IR Spectrometer (Thermo Scientific, Marietta, GA, USA) using a smart multi-Bounce ARK accessory (Thermo Nicolet) equipped with a calcium fluoride crystal. Aβ25-35 peptide solution (incubated after 12 h in tube) was transferred onto the crystal for analysis. A background spectrum was subtracted from all the samples’ spectra. Spectra were acquired in the 4,000 cm^−1^ to 400 cm^−1^ with a spectral resolution of 4 cm^−1^ over 64 scan. The experiment was performed in triplicate.

### ThT Fluorescence Assay

The Hitachi F-4500 fluorescence spectrometer was used to study on the dynamics of amyloid peptide aggregation by ThT assay. A total of sample solution (incubated peptide solution (25 μL), ThT fluorescence solution (25 μL) and solvents (150 μL)) was added to a 0.1 cm quartz cells. ThT fluorescence spectra (excitation at 450 nm, emission at 485 nm) were measured at the following time intervals: 0, 20, 40, 60, 120, 180, 360 and 720 min. The measurement was repeated three times. For the fluorescence, the intensity was the average value of every sample at each time point. The volume ratio of polypeptide solution and ThT (1 mM) was 25:25.

### Size distribution

The size distribution of Aβ25-35 peptide in water was acquired using a laser diffraction size analyzer (MS3000, Malvern). A total of sample solution (incubated peptide solution (200 μL), water (3800 μL)) was added to a 1 cm quartz cells. The measurements were performed at room temperature and all data were obtained with the software provided by Malvern Instruments. The turbidity of Aβ25-35 peptide in water was measured by using Hitachi F-4500 fluorescence spectrometer at room temperature (Em = Ex = 400 nm). A total of sample solution (incubated peptide solution (25 μL), water (175 μL)) was added to a 0.1 cm quartz cells. All the experiment were performed in triplicate.

### Cytotoxicity assay

Human neuroblastoma SH-SY5Y cells were cultured in Dulbecco’s modified eagle medium (DMEM) supplemented with 10% fetal calf serum at 37 °C in a humidified (5% CO_2_, 95% air) atmosphere. The cells were planted in a 96-well microplate at a density of 8000 cells per well for CCK-8 assays. After being cultured for 24 h (the structure of Aβ25-35 peptide incubated in PB and PBS solution was same, seen in Fig S3), the cells were exposed to the different concentration peptide incubated at 37 °C for 24 h and 48 h, and cytotoxicity was assayed using CCK-8 kits (Dojindo Molecular Technologies, Tokyo, Japan). Absorbance was detected at 450 nm with a Tecan Infinite M200 microplate reader (Tecan, Durham, USA). Each experiment was performed in triplicate.

### Hemolytic activity of peptides

Hemolytic activities of the peptides were determined using healthy human erythrocytes. Erythrocytes were prepared by centrifuging 1 mL of fresh blood (1000 × g, 10 min), re-suspending the pelletted cells in 1 mL sterile PBS (pH = 7.4). The cells were washed with PBS three times; in the final wash the cells were re-suspended in 0.75 mL PBS. From this, a 2% erythrocyte suspension was prepared for the assay. Aliquots of sterile water (positive control), peptide, and PBS (negative control) were used in a 24 well plate. 40 μM PBS-Aβ25-35 and Milli-Q water-Aβ25-35 were tested. The assay was then incubated (24 h, 37 °C). After centrifugation (1000 × g, 10 min), aliquots of supernatant were carefully transferred to a 96 well plate and the absorbance was obtained for each well. Percent hemolysis was calculated as previously described^36^.

### Dye Leakage Experiment

The Aβ25-35 aggregates solution are prepared as the described for ATR-FTIR experiments. For each data point, the baseline fluorescence of the empty and dye-encapsulated DOPC vesicles was measured for 30 s. The Aβ25-35 aggregates solution incubated for 12 h was then added into dye-encapsulated DOPC vesicles and they were together incubated for 24 h on the thermo-shaker at 37 °C. After that, the fluorescence was recorded at 30 s. The increase of fluorescence induced by total disruption of the lipid vesicles was measured by the addition of Triton X-100 to a final concentration of 0.05%. The measurement of fluorescence of part disruption of the lipid vesicles was induced by adding peptide aggregates solution. The dye leakage is reported according to the following equation^37^:

$${\rm{Percentage}}\,{\rm{of}}\,{\rm{dye}}\,{\rm{leakage}}=\frac{F{\rm{peptide}}-{\rm{Fbaseline}}}{{\rm{Ftriton}}-{\rm{Fbaseline}}}\times 100$$

All dye leakage experiments were conducted at room temperature(approximately 25 °C) in 10 mM sodium phosphate buffer, pH 7.4. Each experiment was repeated three times for Aβ25-35 aggregates solution.

### Fluorescence experiment

SH-SY5Y cells were seeded on glass coverslips of 14 mm 2 in culture dishes (Nunc, USA) at a density of 1 × 105 cells per well at 37 °C for 24 hours. Before imaging experiments, the growth media was replaced with 2 mL fresh medium containing 40 μM PBS-Aβ25-35 and Milli-Q water-Aβ25-35 labeled by FITC. After incubation at 37 °C for 24 hours, cells were washed three times with PBS solution and were fixed with 4% paraformaldehyde for 10 min. The cells were washed three times with PBS solution. Then the cells were dyed with 1 μg/ml 4′, 6′-diamidino-2-phenylindole (DAPI, Sigma) for 10 min and then the cells were washed three times with PBS solution. The cells were observed with a LSM 710 laser scanning confocal microscope (LSCM, Carl Zeiss LSM710, USA) at 60× magnification. The excitation/emission wavelengths were 488/530 nm for fluorescent peptide and 358/461 nm for DAPI.

### Statistical analysis

The ThT data, CD data, Size distribution data, AFM image data and Cell data were analyzed with OriginPro 8.0 software, JASCO software, Malvern software and NanoScope Aanlysis (1.8) software.

## Electronic supplementary material

Supporting information
