# Supplementary material for: Nanostructural Differentiation and Toxicity of Amyloid-β25-35 Aggregates Ensue from Distinct Secondary Conformation
Source: Sci Rep. 2018 Jan 15;8:765. doi: 10.1038/s41598-017-19106-y (PMC5768673; doi:10.1038/s41598-017-19106-y)
Supplement: Supplementary file 1 — Supporting information [file 41598_2017_19106_MOESM1_ESM.pdf]

Supplementary Information:

Nanostructural Differentiation and Toxicity of Amyloid-β25-35 Aggregates Ensue from Distinct Secondary Conformation

Yongxiu Song[1,3‡], Ping Li[2‡], Lei Liu[1*],Christian Bortolini[3], Mingdong Dong[3*]

[1]Institute for Advanced Materials, Jiangsu University (P. R. China)

[2]National Center for Nanoscience and Technology (NCNST), No. 11, Beiyitiao Zhongguancun, Beijing, P. R. China

[3]Interdisciplinary Nanoscience Center (iNANO), Gustav Wieds vej 14, Building 1590, Aarhus C, Denmark.

* To whom correspondence should be addressed; E-mail: liul@ujs.edu.cn,dong@inano.au.dk

DATE

Supporting figures:

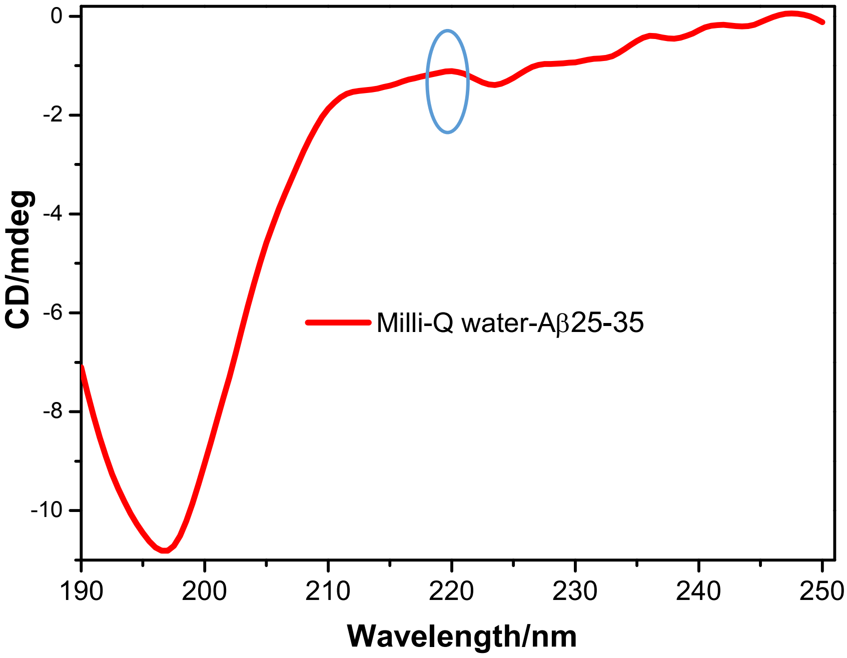

Figure S1 Circular dichroism spectra of Aβ25-35 (100 μM) after 12 h incubation at 37°C in Milli-Q water.

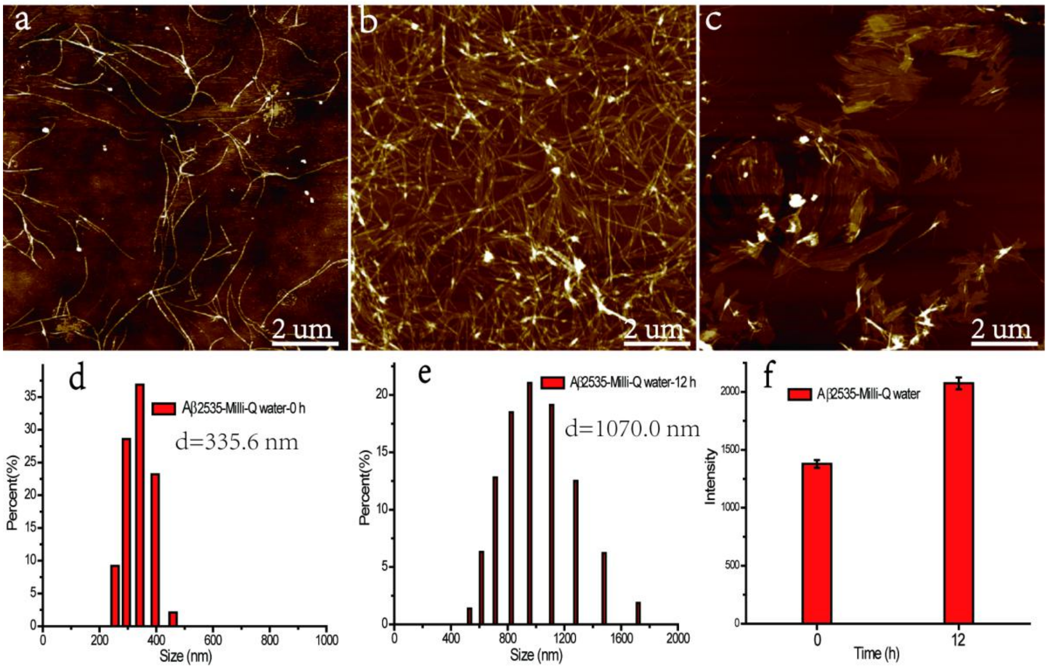

Figure S2 AFM images of Aβ25–35 (100 μM) in Milli-Q water on SiO_2 substrate (a), glass (b) and mica (c) . The size distribution of Aβ25–35 (100 μM) was incubated in Milli-Q water at 0 h (d) and 12 h (e), respectively; (f) Turbidity at 0 h and 12 h of Aβ25–35 aggregates (100 μM) was incubated in Milli-Q water by light scattering

method (Em=Ex=400 nm). Aβ25-35 can self-assemble into fibrils in bulk MQ water and its size distribution in bulk MQ water was increased with increasing incubation time.

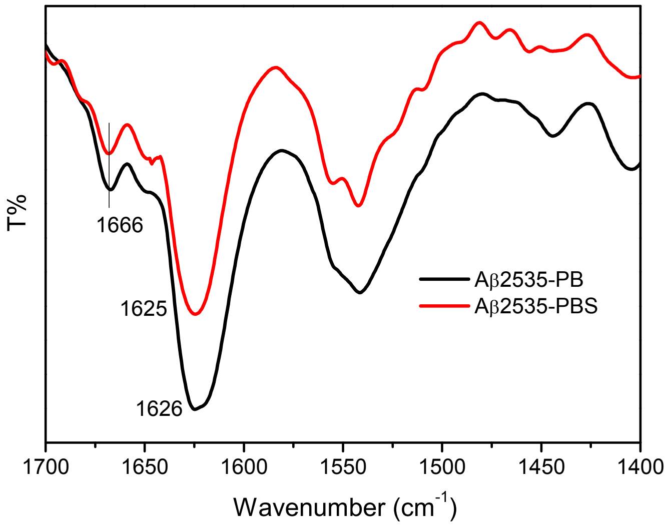

Figure S3 ATR-FTIR spectrum of Aβ25–35 (100 μM) in PB and PBS solution. The structure of Aβ25-35 peptide incubated in PB and PBS was almost same from ATR-FTIR analysis.

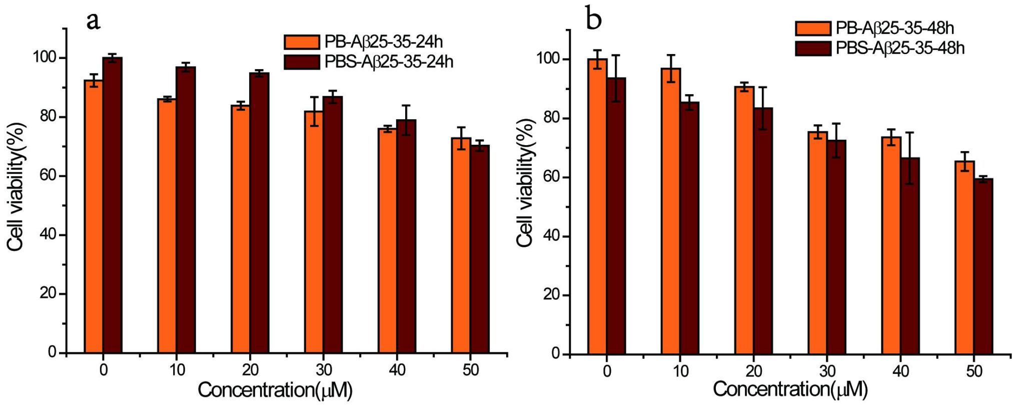

Figure S4 The cell viability of Aβ25–35 aggregates in PB and PBS solution incubated for 24 hours and 48 hours. The cytotoxicity of Aβ25-35 in different solution PB and PBS displayed the similar cell viability.

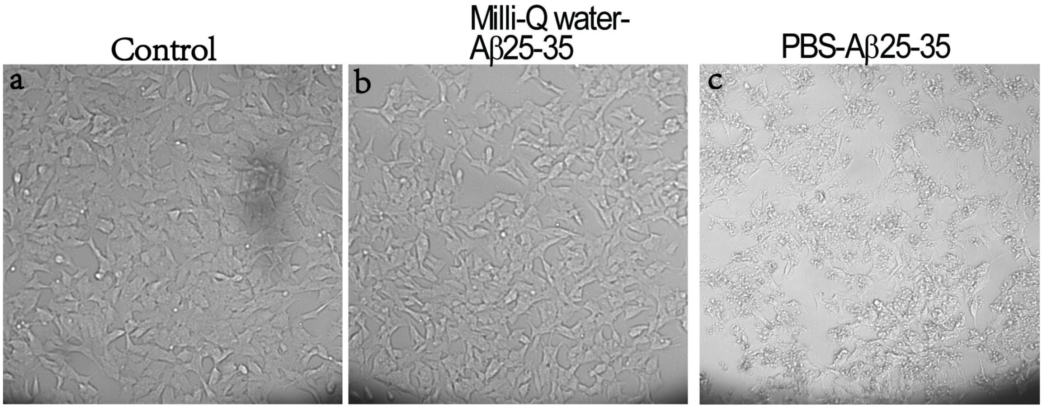

Figure S5 Morphology of the SH-SY5Y cells with or without Aβ25-35 amyloid peptide. (a) Control cells; (b) Control cells were incubated with Aβ25-35 amyloid (40 μM) in Milli-Q water; (c) Control cells were incubated with Aβ25-35 amyloid (40 μM) in PBS buffer. The cells were observed with an IX73 microscope (Olympus, Japan) at 20 times magnification.

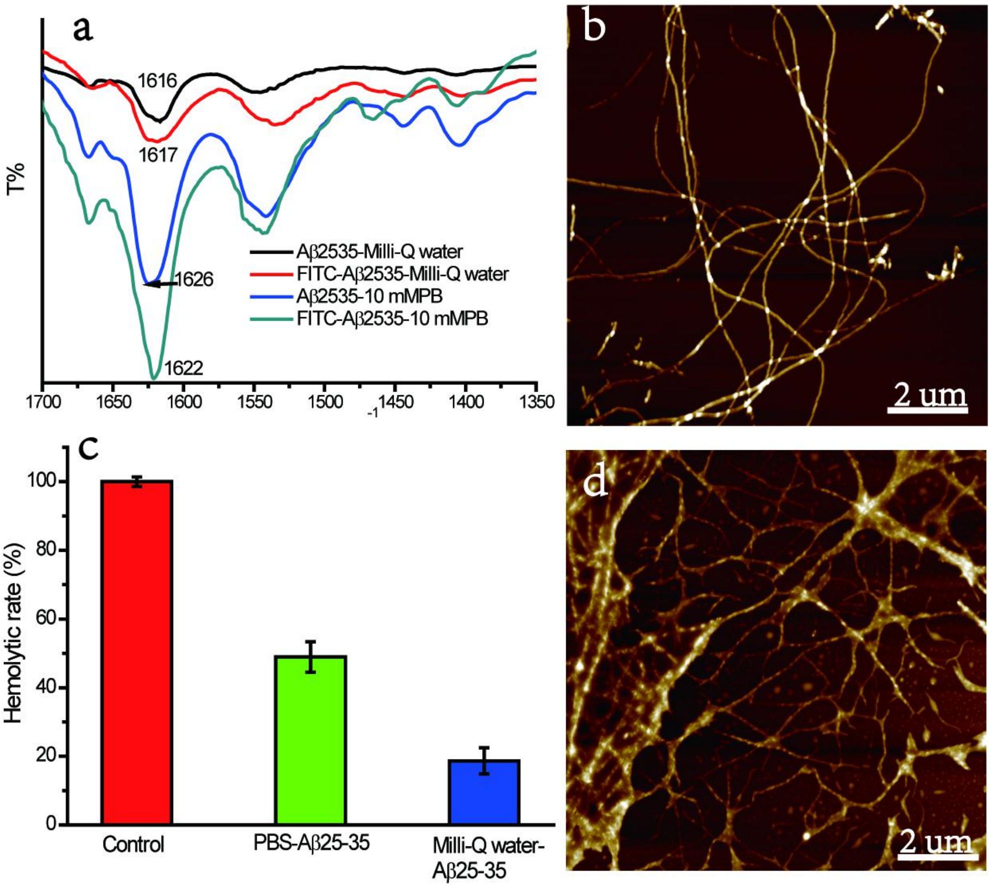

Figure S6 ATR-FTIR spectrum of FITC-Aβ25–35 (100 μM) in Milli-Q water and PB solution(a); AFM images of FITC-Aβ25–35 (100 μM) (after 12 h incubation in Milli-Q water(b) and PB solution(d)) on mica. Percentage release of hemoglobin of healthy human erythrocytes after 24 h incubation with Aβ25-35 in different solutions at 37°C(c). The absorbance peak around at 1617 cm^{-1} and 1622 cm^{-1} of

FITC-Aβ25–35 in Milli-Q water and PB solution are almost same as that of Aβ25–35 and AFM images of FITC-Aβ25–35 aggregates were presented to be typical fibrils, which verify that FITC labeling of Aβ25–35 do not change the nature of the protein. Hemolytic potentials of Aβ25-35 in different solutions were assessed in vitro using freshly isolated erythrocytes obtained by hemolysis experiments. The hemolytic activity of Aβ25–35 was quantified by the release of hemoglobin. Erythrocytes were incubated with 40 μM Aβ25–35 in PBS solution and Milli-Q water at 37°C for 24 h (Fig S6(c)). Under these conditions, Aβ25-35 in PBS buffer caused 48.9 ± 4.4 % hemolytic effect, while Aβ25-35 in Milli-Q water only caused 18.7 ± 3.8% hemolytic activity. Hemolysis assay showed a higher hemolytic effect of Aβ25-35 in PBS solution than that of Aβ25-35 in Milli-Q water.
